# A Digital Smoking Cessation Program Delivered Through Internet and Cell Phone Without Nicotine Replacement (Happy Ending): Randomized Controlled Trial

**DOI:** 10.2196/jmir.1005

**Published:** 2008-11-28

**Authors:** Håvar Brendryen, Filip Drozd, Pål Kraft

**Affiliations:** ^1^Department of PsychologyUniversity of OsloOsloNorway

**Keywords:** Smoking cessation, behavior change, Internet, cell phone, interactive voice response, short message service

## Abstract

**Background:**

Happy Ending (HE) is an intense 1-year smoking cessation program delivered via the Internet and cell phone. HE consists of more than 400 contacts by email, Web pages, interactive voice response, and short message service technology. HE includes a craving helpline and a relapse prevention system, providing just-in-time therapy. All the components of the program are fully automated.

**Objective:**

The objectives were to describe the rationale for the design of HE, to assess the 12-month efficacy of HE in a sample of smokers willing to attempt to quit without the use of nicotine replacement therapy, and to explore the potential effect of HE on coping planning and self-efficacy (prior to quitting) and whether coping planning and self-efficacy mediate treatment effect.

**Methods:**

A two-arm randomized controlled trial was used. Subjects were recruited via Internet advertisements and randomly assigned to condition. Inclusion criteria were willingness to quit on a prescribed day without using nicotine replacement and being aged 18 years or older. The intervention group received HE, and the control group received a 44-page self-help booklet. Abstinence was defined as “not even a puff of smoke, for the last seven days” and was assessed by means of Internet surveys or telephone interviews 1, 3, 6, and 12 months postcessation. The main outcome was repeated point abstinence (ie, abstinence at all four time points). Coping planning and self-efficacy were measured at baseline and at the end of the preparation phase (ie, after 2 weeks of treatment, but prior to cessation day).

**Results:**

A total of 290 participants received either the HE intervention (n=144) or the control booklet (n=146). Using intent-to-treat analysis, participants in the intervention group reported clinically and statistically significantly higher repeated point abstinence rates than control participants (20% versus 7%, odds ratio [OR] = 3.43, 95% CI = 1.60-7.34, *P* = .002). Although no differences were observed at baseline, by the end of the preparation phase, significantly higher levels of coping planning (*t*_261_ = 3.07, *P* = .002) and precessation self-efficacy (*t*_261_ = 2.63, *P* = .01) were observed in the intervention group compared with the control group. However, neither coping planning nor self-efficacy mediated long-term treatment effect. For point abstinence 1 month after quitting, however, coping planning and self-efficacy showed a partial mediation of the treatment effect.

**Conclusions:**

This 12-month trial documents a long-term treatment effect of a fully automated smoking cessation intervention without the use of nicotine replacement therapy. The study adds to the promise of using digital media in supporting behavior change.

## Introduction

Two reviews [1,2] of a total of 29 randomized controlled trials (RCTs) of computer-based interventions for smoking cessation testify to the effectiveness of this form of intervention. However, as evidenced by these reviews, our knowledge on what differentiates successful interventions from unsuccessful interventions remains limited. Insufficient reporting of the interventions may have contributed to the difficulty to identify patterns of predictors for intervention efficacy [2]. Hence, in the following section we first describe the rationale behind the intervention under scrutiny, before formulating the aims and hypotheses of the study. Designing a complex smoking cessation intervention requires a multitude of choices to be made. By pointing to some key principles and assumptions that guided us in designing Happy Ending (HE), we hope to convey some information not only about whether this intervention worked, but also about why it may have worked.

### The Theory and Research Behind Happy Ending

The psychological processes that quitters experience are different across various time points and follow a certain chronology [3-15]. Consequently, smoking cessation interventions should follow the same chronology, and the program content should be organized according to the psychological processes that people experience at certain time points. It is difficult to achieve this adjustment with a static and hierarchically organized Web page. One way to solve this in practice is to organize the program content into multiple pieces that are made available to the client sequentially and for a restricted period. In this way, the client progresses through a predetermined sequence of modules (ie, iterations) where the degrees of freedom are restricted. This can be referred to as tunneling [16], and it is the core organizing principle of HE.

HE starts with a 14-day preparation phase. Every morning, the client receives an email containing a hyperlink. By activating the link, the smoker has access to that particular day’s website. See Table 1 for details of the number of contact points and their distribution over the program period. The order of the websites was based on a reasoned chronology, modeled according to psychological processes that people experience at certain time points in a process of therapy-supported self-regulation [3-15]. The first days were constructed to establish confidence in the treatment provider and a therapeutic alliance between the provider and receiver of the treatment [17]. Additionally, a major focus was to ensure that the client understood that self-awareness, self-monitoring, active participation, and engagement are crucial ingredients for personal goal attainment [18,19].

The participant is educated about his or her psychological profile and responses, both as a person and as a smoker. Consequently, smokers will be more aware of, and will learn about, such things as their smoking behavior and nicotine dependence, reasons for previous failures to quit, motivational basis for quitting, general and task-specific self-efficacy, problems that people often experience when quitting, and stress and weight regulation. One of the most important predictors of the outcome of self-change processes is self-efficacy, or the extent to which the person is confident that he or she will succeed [20]. Precessation and postcessation self-efficacy have been shown to play important roles in smoking cessation [21]. Consequently, HE is constructed to instill a high but realistic level of self-efficacy in the participants.

A crucial ingredient of the program is to educate the participants about the cognitive, affective, and behavioral reactions that smokers usually experience if a slip occurs (ie, if they smoke some cigarettes during the quit attempt). In HE, participants are told that the administrators expect that most of them will experience one or more slips [8]. Participants are told that it is not critical whether they experience a slip, but rather, how they react emotionally and behaviorally to slips. Hence, we try to prevent the devastating cognitive and emotional consequences (“snowballing”) of breaking zero-tolerance rules [19]. By being prepared for these reactions, being able to recognize them when they occur, and having specific skills and support systems to master such setbacks, the probability that the self-regulation process will be successful increases significantly [19].

Furthermore, we have applied principles from cognitive behavioral therapy [22]. A core assumption here is that the client will learn to master his or her own life problems (ie, solve problems and difficult situations) without smoking. To do this successfully, the client must be able to recognize, understand, and change inappropriate patterns of thought that occur in relation to the acute problems that are experienced. HE attempts to instill this capability by giving the participants small practical problems to solve (behavioral tasks) or some issue to consider (cognitive and emotional tasks). Then, on the following day, the participants are asked to write down notes related to the previous day’s issue in an interactive diary. The preparation phase also contains elements of behavioral skills training. These consist of (1) techniques related to the acquisition of new skills, such as self-stopping, the use of substitutions, self-monitoring, and foresight [19], and (2) coping planning [23] related to high-risk relapse situations.

In addition to the activities that take place on the websites, the participants stay in touch with HE via short message service (SMS) text messaging and interactive voice response (IVR). The purpose of this is twofold. First, it is important that the participants become used to communicating with HE via the cell phone because it plays a crucial role in the rest of the program. Second, the cell phone is used to support the other activities and processes that are initiated via the websites.

After the preparation phase comes a 30-day active quitting phase, which is initiated with the actual cessation attempt. Here, a number of activities are included to ensure that participants are actively involved in their own attempt to quit. Hence, there are numerous contact points every day between the participant and HE. Participants receive an email in the morning with a link to that day’s specific website. However, there are several differences between these websites and the ones in the preparation phase. First, the Web activities focus on the motivational conflict that many smokers will experience during the first smoke-free days. Along with the temptations and impulses to smoke, this motivational conflict implies that the effect of the expected consequences of smoking versus not smoking tends to change. In short, the positive short-term consequences of smoking (eg, feeling more relaxed, less irritable) tend to be inflated, while the long-term negative consequences of smoking (eg, health problems) seem to be deflated during the first days and weeks of a quit attempt [4,10,19]. To prevent this, the participants receive IVR messages about the short-term positive consequences of their quitting. This information resembles a type of biofeedback (eg, “Today your blood pressure has been reduced to that of a nonsmoker.”), and the topic is further elaborated on the website of the day. The IVR messages are received every morning in the active quitting phase when the client logs on to the program by calling HE. The message also informs the client that he or she can read more about this topic on the website of the day. If the quitter does not log on, several reminders will be automatically activated by the program. Another purpose of this log-on procedure is to ensure that the quitter is actively involved, self-aware, and self-monitoring.

The websites in the active quitting phase contain elements and activities collected from social cognitive learning theory [20] and self-regulation theory [9,24]. Particular emphasis is placed on the importance of postcessation self-efficacy [20], identified as a key predictor of the outcome of a smoking cessation attempt [21]. In this regard, two types of self-efficacy expectations are important: the general expectation that one will successfully quit (success expectations), and the expectancy that one can manage difficult situations (temptations) without smoking. A major aim of the program is to strengthen the participants’ postcessation self-efficacy by preparing them for tempting situations (ie, cognitions and emotions that they will experience), helping them learn from mastery experiences, and reminding them that they have a number of tools to help overcome the craving. Moreover, the client is encouraged to make concrete implementation intentions and coping plans regarding how to stay smoke-free in the immediate future [23,25]. Finally, every day the quitter continues to follow activities related to the diary: reading, considering, performing, and writing. In this phase, many of the tasks are based on principles from cognitive behavioral therapy and behavioral skills learning (eg, problem-focused mastery and self-stopping) [20,26].

An effective program should take into account the fact that a large proportion of quitters are likely to relapse. Relapses typically follow a pattern of intermittent episodes of smoking more often than they follow an abrupt resumption of smoking [7]. Hence, in most cases, a relapse has been preceded by one or more lapses, and one or more lapses clearly increase the risk of a full-blown relapse [7]. Among those who experience a first lapse, a subsequent lapse or relapse is very likely to occur, often within 1-4 days [3,12]. For intervention purposes, two lessons seem relevant. The first, addressed in almost all smoking cessation interventions, is the prevention of the occurrence of general risk factors. Second, programs that offer just-in-time therapy to remove or prevent escalation of processes that increase the risk of subsequent relapse are likely to be more effective. Moreover, such an intervention should aim at reducing the number of cigarettes smoked during the slip because this variable seems to predict the probability of later abstinence [27]. One way to shorten the period of smoking and reduce the amount smoked would be to have the client who slips prepare an implementation intention [28] regarding how and when to resume the quit attempt (eg, “I will continue my quit attempt from tomorrow morning.”). Consequently, an automated IVR-based relapse prevention system is incorporated in HE. It entails the participant being called by HE every night (the logging-off procedure). The quitter is then asked whether he or she has smoked during the day. If the participant has smoked during the day (reported by pressing 2), this will activate a therapy regimen (ie, 1 of 5 different regimens depending on how many slips the quitter has previously reported). The purpose of the regimen is to induce the participant to attribute the slip to situational factors, thereby preventing negative emotions and a full-blown relapse. Furthermore, an important element is to make the quitter accept that if he or she relapses to smoking, it is part of a deliberate decision and not something that the person is more or less powerless to prevent.

The quitter may experience close-call situations in which the ex-smoker is brought to the brink of smoking [12,13], at which the occurrence of smoking or nonsmoking seems to be influenced by the quitter’s acute coping responses. To help participants cope with close-call situations, HE contains an IVR-based craving helpline. Participants are instructed to call the helpline every time they are tempted to have a cigarette (making use of the principles of implementation intention and coping planning). Upon calling, they are asked to report how they feel and thus what kind of help they need. By the push of a button, clients choose between (1) emotion regulation, (2) motivation boost, and (3) stress regulation. Next, the client will hear a therapeutic message specifically designed to solve his or her problem (a new message at each call).

Finally, HE offers an 11-month follow-up phase. During this phase, the logging-off procedure continues daily for another 4 weeks, twice a week for another 2 weeks, and then once a week for the remaining follow-up period. Hence, the system will register slips and activate the relapse prevention system for the whole period. Furthermore, the participants have access to the craving helpline during the whole follow-up phase. Finally, the quitter receives a number of encouraging SMS and IVR messages during this phase.

In summary, compared with most other digital smoking cessation programs [1,2,29-32], HE has some unique features. First, it combines four media approaches: email, Web, IVR, and SMS. Second, HE is distinct in relying on tunneling [16] as a broad structuring principle. Finally, HE includes two components of just-in-time therapy (ie, the craving helpline and the relapse prevention system), which are not yet commonly observed in the field [33].

### Previous Trials

We previously investigated the same digital multimedia smoking cessation intervention using a similar design in an earlier 12-month RCT [29]. Before that trial, only short-term effects (ie, 3 months after quitting) of digital cessation interventions were documented [30-32]. Thus, the trial represented a significant contribution to the potential of applying digital media in smoking cessation interventions. The study, however, had two important shortcomings, which are addressed in the current trial.

First, in the previous trial [29], nicotine replacement therapy (NRT) was part of the recruitment inducement. In the final sample, 9 out of 10 subjects, in both experimental conditions, used NRT during their quit attempt. Consequently, it could be that the results only applied to those willing to use NRT, and, hence, there might have been a problem with generalizing the findings to all smokers. Therefore, in the current trial we aimed to recruit subjects who were willing to quit without the adjacent use of NRT.

Second, the previous trial failed to document the mediation effect of the program on relevant psychological variables. Technically, a complete mediation effect was found [29] on self-efficacy at 1 month after smoking cessation, but it was not possible to conclude this from the analysis because of the confounding variable of smoking status. One way to avoid this confounding variable is to investigate effects obtained before cessation, which lead us to the third aim of the current study: to explore the psychological effects caused by the intervention and eventual mediation of treatment effect related to these variables.

### Hypotheses

We tested the hypothesis that a digital, fully automated smoking cessation intervention would produce an increased 12-month abstinence rate compared with a control condition of a self-help booklet. Furthermore, we expected the digital intervention to increase precessation levels of coping planning and self-efficacy. Finally, we expected the hypothesized increase in precessation coping planning and self-efficacy to partially mediate the treatment effect.

## Methods

### Design

This was a two-arm randomized controlled trial. Subjects were randomized to either receive HE (intervention), or a 44-page self-help booklet (control), described in further detail below. The trial was registered and approved by the Regional Ethics Committee, Norway, South-East (project number: 2.2005.353).

### Subjects

Subjects were recruited by means of online banner advertisements in Norwegian regional newspapers from February 6 to 10, 2006. Banners were displayed 947,059 times, resulting in 2595 hits, which gave a hit rate of 0.3%. When clicking on a banner, potential subjects were routed to a website containing study information, an informed consent, and a baseline questionnaire. During the informed consent process, participants were informed that they would be arbitrarily split into groups that would receive different tools for smoking cessation. It was specified that the various tools did not include any form of medication and that participation in the study did not require attendance at face-to-face meetings or consultations. However, no information was provided whatsoever about the intervention conditions. Inclusion criteria were 1) willingness to quit on March 6, 2006, 2) at least 18 years old, 3) currently smoking five cigarettes or more on a daily basis, 4) willingness to quit without using NRT, 5) owning a mobile phone, 6) a Norwegian-registered phone number and postal address, and 7) having daily access to the Internet and email.

There were 427 unique registrations, 23 of which did not fulfill the inclusion criteria. Another 82 subjects were excluded because of missing values, and 19 subjects were excluded because they were suspected to know each other, based, for example, on sharing or having the same family name, postal address, email, IP address, or worksite. This was done to reduce the risk of communication across experimental conditions. Finally, seven subjects were excluded randomly because the required number of participants was 296 (according to a power analysis).

### Intervention and Control Conditions

The control group received a 44-page self-help booklet issued by the Norwegian Directorate for Health and Social Affairs. The booklet contains general cessation information, a quit calendar, a 10-day quit log, the phone number of the national quitline, and links to relevant and open online tobacco cessation resources. The booklet recommends 10 days of preparation prior to quitting, in which readers are encouraged to map their smoking habits in the quit log. Additionally, for each of the 10 preparation days, the booklet suggests an exercise aimed at raising awareness about personal smoking habits. The 48-day quit calendar is composed of small, encouraging daily messages about improvements in health and well-being after quitting (eg, “Your risk of cardiovasculardisease is reduced.” and “Does food taste better to you now?”).

The treatment group received the digital multimedia intervention HE, described above. See Table 1 for details on the number of contact points and their distribution over the program period. All contacts were automated.

**Table 1:** Overview of potential contact points between HE and user during the entire intervention period ^a^

Component of HE	Week 1-2							Week 3-6							Week 7-8 ^b^							Week 9-10							Week 11-15							Week 16-54						
Email	∙	∙	∙	∙	∙	∙	∙	∙	∙	∙	∙	∙	∙	∙																												
Web page	∙	∙	∙	∙	∙	∙	∙	∙	∙	∙	∙	∙	∙	∙																												
Text message	∙	∙	∙	∙	∙	∙	∙	∙	∙	∙	∙	∙	∙	∙	∙	∙	∙	∙	∙	∙	∙	∙	∙	∙	∙	∙	∙	∙			∙			∙				∙				
Log-on call								∙	∙	∙	∙	∙	∙	∙	∙	∙	∙	∙	∙	∙	∙																					
Log-off call								∙	∙	∙	∙	∙	∙	∙	∙	∙	∙	∙	∙	∙	∙	∙	∙	∙	∙	∙	∙	∙				∙			∙							∙

^a^ The seven columns within the week correspond to the number of days in a week. Each dot represents one intended contact.

^b^ The number of messages per day was gradually reduced from 3 to 1 over the span of these 2 weeks.

#### Randomization, Allocation, and Data Collection Procedure

Based on computer-generated random digits, 296 subjects were randomly allocated to either the HE intervention or the booklet control condition. Stratified block randomization was applied to ensure equal numbers of both males and females in each group. Randomization was performed by the experimenter. The names and identities of the subjects, however, were concealed to the experimenter during randomization. After randomization, subjects received an email informing them which tool they would be provided with and when and how they would receive it. Subjects in the HE group were told that the intervention would begin on February 20, 2006, but that the designated quit date was March 6, 2006. Subjects in the control group were told about the booklet and were encouraged to read the booklet thoroughly before the designated quit date and to use it actively throughout their quit attempt. Information on the type of treatment provided to the other group was withheld for subjects in both experimental conditions.

Data were collected by means of online questionnaires at baseline, precessation, and at 1, 3, 6, and 12 months after cessation. An email containing a link to the questionnaire was sent to the subjects at each data collection point. Two email reminders were sent to nonresponders. For all postcessation follow-ups, telephone interviews were conducted with subjects who had not responded after the second reminder. The telephone interviews were structured and standardized with no person-to-person counseling or face-to-face contact between experimenters and subjects at any point. Four attempts were made to contact nonresponders by telephone in both conditions at every data collection point.

#### Variables

Abstinence was defined as having been completely smoke-free for the past 7 days. Subjects with missing values on abstinence data were coded as smokers. Abstinence data were based on self-reports with no biochemical verification and were assessed at 1, 3, 6, and 12 months after cessation. The main outcome in this trial was repeated point abstinence, that is, abstinence on all four postcessation measuring points.

Nicotine dependence was assessed by the Fagerström Test for Nicotine Dependence (FTND) [34] (Cronbach alpha .68). Self-efficacy was measured using two items rated on a 7-point scale and averaged (Cronbach alpha .82). Coping planning was measured using five items rated on a 4-point scale. Coping planning refers to behavioral and cognitive strategies used to connect anticipated barriers with suitable coping responses [23] (Cronbach alpha .86). Program adherence was continuously and automatically registered by a computer during the trial; that is, each and every user-initiated activity on the Web and the IVR service was registered.

The present study intended to evaluate the effect of HE without the adjunct use of NRT. All eligible candidates for the study were informed about this and agreed to attempt quitting without using NRT. However, it is important to note that subjects received information and recommendations regarding NRT in both conditions. For technical reasons, it was not possible to modify this feature from the program or the booklet. Therefore, to be able to control for possible NRT use, the subjects were asked at 3 months whether they had used NRT to quit smoking.

#### Data Analysis

An alpha level of .05 was chosen for all statistical tests and all tests were two-tailed. To check for differences between experimental conditions at baseline, *t* tests were used for scales and chi-square tests were performed for categorical data. Furthermore, all chi-square tests based on 2 x 2 contingency tables were applied the Yates continuity correction. Outcomes were examined using the intent-to-treat principle (ie, missing was counted as smoker).

For repeated point abstinence at 12 months and for point abstinence at 1, 3, 6, and 12 months after cessation, the odds ratio (OR) with the 95% confidence interval (CI) and a chi-square test for experimental condition were carried out, respectively. Hierarchical logistic regression was applied [35] to test whether coping planning and self-efficacy mediated the effect from the experimental condition on abstinence. These analyses were based on a complete case approach.

## Results

### Program Use, Attrition, and Subject Characteristics

**Figure 1:**
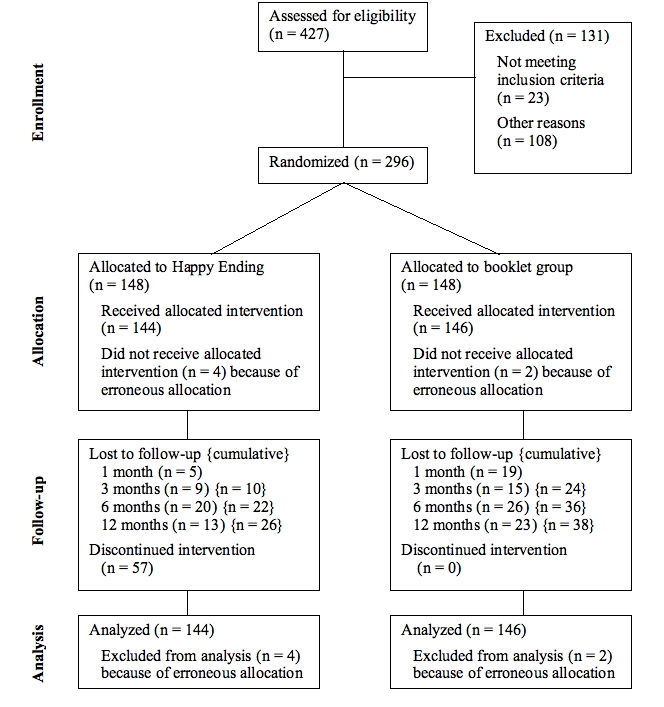
Flowchart of participants

The flow of participants is depicted in Figure 1. Six of the 296 subjects were excluded after randomization because it was discovered that they did not fulfill the inclusion criteria: two were signed up by another person and hence did not intend to quit, and four reported already having quit smoking at the point of randomization. These subjects are referred to erroneous allocations in Figure 1. Consequently, the final number of participants was 290. Cumulative loss (loss to follow-up on at least one of the previous follow-ups) is shown in curly brackets. Also note that participants who discontinued the intervention were approached for data collection.

At baseline, there were no variables on which treatment and control subjects differed significantly (Table 2).

**Table 2:** Baseline sample characteristics^a^

Characteristic	HE (n = 144)	Control (n = 146)
Female, No. (%)	72 (50)	73 (50)
Has a college degree, No. (%)	70 (49)	76 (52)
Age (years)	39.5 ± 11.0	39.7 ± 10.8
Nicotine Dependence (FTND)	4.5 ± 2.3	4.6 ± 2.2
Cigarettes smoked per day	16.6 ± 7.2	17.6 ± 7.0
Precessation self-efficacy	5.1 ± 1.4	5.1 ± 1.3
Precessation coping planning	2.3 ± 0.6	2.4 ± 0.7

^a^ Numbers are mean ± SD except where noted.

Computerized logging routines revealed that subjects in the treatment condition, to a large extent, accomplished the actions intended in the program design (ie, in 5-6 out of 10 cases). See Table 3 for details of program adherence, and Table 1 for details of contact points. Few clients, however, used the craving helpline: 80 (56%) never called the helpline, 45 (31%) called once or twice, and 19 (13%) called three times or more.

**Table 3:** Mean number of active client actions for three components of HE (n = 144)^a^

Active Client Action	Range	Mean	SD	%
Log-on call	0-42	26	16	62
Opening Web page	0-44	26	13	59
Responding to log-off call	0-102	53	37	52

^a^ The log-off call was initiated by the program. Here, responding means answering either “yes” or “no” to the abstinence question. Theoretical range and observed ranges coincide with one exception: theoretical maximum for log-off calls is 104. The right-hand column shows the average percentage of actions completed.

In total, 57 subjects discontinued the intervention, of which 36 did so during the first 6 weeks. The reason for dropout was not recorded. These subjects were approached by Web and telephone interviews in exactly the same way as were program participants and subjects in the control group. At 1 month, 17 subjects (12%) reported that they found HE “not at all helpful,” 74 subjects (51%) found HE to be “helpful,” and 46 subjects (32%) reported HE to be “very helpful”; data were missing for 7 subjects (5%).

As shown in Table 4, the response rates observed in this study were generally high across both the experimental condition and across time. The response rate to the Web survey fell more sharply than total response rate over time. Nonresponders to Web surveys were approached by telephone. Correspondingly, the proportion of responses gathered by means of telephone interviews increased, suggesting the importance of combining Web surveys with telephone interviews, particularly for long-term follow-up. At 1 month after cessation, significantly more subjects in the treatment condition than the control condition responded to surveys (*χ* ^2^_1_ = 7.5, *P* = .006). Hence, selective attrition is a problem regarding point abstinence at 1 month. Between-group differences regarding total response rate at preparation, 3, 6, and 12 months, however, were not significant. The cumulative dropout rate at 12 months (ie, loss to follow-up at 1, 3, 6, and 12 months) did not significantly differ between treatment and control conditions. Hence, selective attrition was not a problem for interpretation of 12-month repeated point abstinence.

**Table 4:** Number of Web, phone, and total responses across conditions at specified time points; HE (n = 144) and control (n = 146)

Time	------ Web ------		----- Phone -----		----- Total -----	
	HE	Control	HE	Control	HE	Control
Preparation	132	131	–	–	132	131
1 month	128	119	11	8	139	127
3 months	119	110	16	21	135	131
6 months	101	97	23	23	124	120
12 months	101	89	30	34	131	123

### Abstinence

The main finding from this trial was that participants in the intervention condition (n = 29, 20%) reported clinically and statistically significantly higher repeated point abstinence rates than control participants (n = 10, 7%) (OR = 3.43, 95% CI = 1.60-7.34, n = 290, *P* = .002). Hence, HE was efficacious in helping smokers to achieve long-term abstinence. HE was equally effective across sample subgroups, as defined by sex, age, and nicotine dependence; no interaction effect between experimental condition and any baseline characteristic was found.

Despite agreeing to quit without using NRT, 34 subjects (24%) in the treatment condition and 14 subjects (10%) in the control condition reported NRT use. The proportion of NRT users was significantly higher in the treatment condition compared with the control condition (*χ* ^2^_1_ = 9.3, *P* = .002). When adding NRT use along with experimental condition in a logistic regression model, the OR decreased to 2.86 (95% CI = 1.31-6.24, n= 290, *P* = .008). In summary, our hypothesis that HE would produce an increased abstinence rate, compared with a control group receiving a self-help booklet, was supported, even when controlling for NRT use.

Table 5 shows the point abstinence and repeated point abstinence rate for each of the four follow-ups. Abstinence rates were significantly higher for the treatment condition than the control condition at 1, 3, and 6 months. At 12 months, however, the difference only reached a marginal significance level. Moreover, there is reason to believe that the effect size reported for 1-month abstinence is inflated because of selective attrition. Note from Table 5 that the proportion of abstainers gradually decreases from 1-6 months, but in fact increases from 6-12 months, particularly in the control condition. Hence, the lack of significant difference between groups at the 12-month point was, for the most part, due to subjects in the control condition performing a second quit attempt and not so much that subjects in the treatment condition relapsed to smoking.

**Table 5:** Point abstinence and repeated point abstinence rates across conditions at specified time points

Time After Cessation		HE (n = 144), No. (%)	Control (n = 146), No. (%)	OR	95% CI	*P*
**Point Abstinence ^a^**						
	1 month	60 (42)	25 (17)	3.46	2.01-5.95	.001
	3 months	51 (35)	23 (16)	2.93	1.67-5.14	.001
	6 months	42 (29)	20 (14)	2.59	1.43-4.69	.002
	12 months	47 (33)	33 (23)	1.66	0.99-2.79	.07
**Repeated Point Abstinence**						
	1 + 3 months	43 (30)	17 (12)	3.23	1.74-6.00	.001
	1 + 3 + 6 months	34 (24)	10 (7)	4.24	1.99-8.89	.001
	1 + 3 + 6 + 12 months	29 (20)	10 (7)	3.43	1.60-7.34	.002

^a^ Point abstinence was based on 7-day point prevalence and intent-to-treat.

### Precessation Coping Planning and Self-Efficacy

Pearson *r* between baseline and precessation coping planning was .32 (*P* < .001). The level of precessation coping planning was significantly higher in the treatment condition (mean = 3.0, SD = 0.5) than the control condition (mean = 2.8, SD = 0.5; *t*_261_ = 3.1, *P* = .002), as hypothesized.

Pearson *r* between baseline and precessation self-efficacy was .54 (*P* < .001). The level of precessation self-efficacy was significantly higher in the treatment condition (mean = 5.5, SD = 1.2) than the control condition (mean = 5.1, SD = 1.3; *t*_261_ = 3.0, *P* = .003), as hypothesized.

The between-group difference for both coping planning and self-efficacy was small, at only one-third of a standard deviation. Coping planning and self-efficacy were tested formally [35] as mediators of treatment effect. Experimental condition, baseline coping planning, and baseline self-efficacy were entered in block one; precessation coping planning was entered in block two; and precessation self-efficacy was entered in block three. Point abstinence at 1 month was the dependent variable. Precessation coping planning showed a small but significant mediation effect in block two, and precessation self-efficacy showed a small but significant mediation effect in block three. In block three, precessation coping planning no longer predicted abstinence significantly, meaning that the increase in precessation coping planning could not add more explanatory power over precessation self-efficacy. The correlation between coping planning and self-efficacy was lower at baseline (*r* = .26, *P* < .001) compared with precessation (*r* = .49, *P* < .001). When the above mediation analysis was repeated with repeated point abstinence at 12 months as the dependent variable, there were no mediation effects whatsoever.

In summary, HE slightly increased the level of both coping planning and self-efficacy during the 2-week preparation phase of the program. The increase in self-efficacy could explain at least some of the initial success in gaining abstinence (ie, at 1 month after cessation).

### Ancillary Analysis

A complete case analysis showed the repeated point abstinence rate at 12 months to be 25% (29/118 subjects) in the treatment group versus 9% (10/108 subjects) in the control group (*χ* ^2^_1_ = 8.22, OR = 3.19, 95% CI = 1.47-6.92, n = 226, *P* = .004). Compared with the intent-to-treat analysis, this represents a small increase in abstinence rate for both groups, but a small decrease in effect size.

We also looked into what happened when subjects who did not use the intervention at some minimal level were excluded. Excluding subjects who performed fewer than five actions in each of the three categories of log-on calls, opening Web pages, and answering log-off calls resulted in an abstinence rate in the treatment condition of 26% (n= 111). Inclusion of only those who used the intervention at some minimum level and applying a complete case approach further increased the quit rate to 29% (n = 100).

## Discussion

This trial demonstrated the efficacy of the digitally delivered and fully automated HE smoking cessation intervention over a self-help booklet condition—without the combined use of NRT—in producing increased repeated point abstinence at 12 months. The ability of HE to increase precessation self-efficacy could explain some success in gaining early abstinence.

The fact that some quitters used NRT, even though they had promised not to do so, resulted in a somewhat inflated effect size. However, this could not seriously compromise conclusions because the main effect from the experimental condition is still clinically and statistically significant even after controlling for NRT use. Hence, the success of HE can be explained by the psychological support provided by the program. Exactly what mechanisms are at play to cause the treatment effect is not fully clear at this stage. We do know that HE instilled a somewhat higher level of precessation self-efficacy compared with the control condition and that this could explain at least some of the initial success in gaining abstinence.

In a previous trial on the same intervention and with a similar design [29], NRT was part of the recruitment inducement, which potentially could have influenced the representativeness of the sample; that is, the results from that trial may apply only to smokers willing to use NRT. In contrast, the current trial recruited smokers willing to quit without the use of NRT. Although some of the subjects used NRT anyway, the treatment effect on the main outcome was still impressive after controlling for NRT use. Hence, this trial significantly adds to the generalizability of the findings in both trials; findings now apply to both NRT users and nonusers. However, generalizability may still be a concern in both trials because of recruitment by self-selection.

This trial could not biochemically verify self-reported claims of abstinence due to the geographic spread of the sample, cost, and other practical concerns. However, false reporting is considered to be minimal when there is little or no personal contact between treatment provider and subjects [36]. In the current trial, the amount of personal contact between experimenters and subjects was equal in both conditions and was restricted to data collection (ie, telephone follow-up of nonresponders); hence, it not likely that misreporting could compromise conclusions.

In summary, this trial extends the public health significance of digital multimedia interventions for smoking cessation. It shows that psychological support can be effectively mediated through modern distance communication technology and that automated support as a stand-alone intervention is, in fact, sufficient for a significant effect on long-term behavior change.

## Abbreviations

FTND: Fagerström Test for Nicotine Dependence
HE: Happy Ending
IVR: interactive voice response
NRT: nicotine replacement therapy
RCT: randomized controlled trial
SMS: short message service
